# Point detection through multi-instance deep heatmap regression for sutures in endoscopy

**DOI:** 10.1007/s11548-021-02523-w

**Published:** 2021-11-08

**Authors:** Lalith Sharan, Gabriele Romano, Julian Brand, Halvar Kelm, Matthias Karck, Raffaele De Simone, Sandy Engelhardt

**Affiliations:** 1 Department of Internal Medicine III, Group Artificial Intelligence in Cardiovascular Medicine, Heidelberg University Hospital, 69120 Heidelberg, Germany; 2 Department of Cardiac Surgery, Heidelberg University Hospital, 69120 Heidelberg, Germany

**Keywords:** Point detection, Mitral valve repair, Endoscopy

## Abstract

**Purpose:**

Mitral valve repair is a complex minimally invasive surgery of the heart valve. In this context, suture detection from endoscopic images is a highly relevant task that provides quantitative information to analyse suturing patterns, assess prosthetic configurations and produce augmented reality visualisations. Facial or anatomical landmark detection tasks typically contain a fixed number of landmarks, and use regression or fixed heatmap-based approaches to localize the landmarks. However in endoscopy, there are a varying number of sutures in every image, and the sutures may occur at any location in the annulus, as they are not semantically unique.

**Method:**

In this work, we formulate the suture detection task as a multi-instance deep heatmap regression problem, to identify entry and exit points of sutures. We extend our previous work, and introduce the novel use of a 2D *Gaussian* layer followed by a differentiable 2D spatial *Soft-Argmax* layer to function as a local non-maximum suppression.

**Results:**

We present extensive experiments with multiple heatmap distribution functions and two variants of the proposed model. In the intra-operative domain, Variant 1 showed a mean $F_1$ of $+ 0.0422$ over the baseline. Similarly, in the simulator domain, Variant 1 showed a mean $F_1$ of $+ 0.0865$ over the baseline.

**Conclusion:**

The proposed model shows an improvement over the baseline in the intra-operative and the simulator domains. The data is made publicly available within the scope of the MICCAI AdaptOR2021 Challenge https://adaptor2021.github.io/, and the code at https://github.com/Cardio-AI/suture-detection-pytorch/.

## Introduction

Mitral valve repair is a surgery of the mitral valve of the heart that seeks to restore its function by reconstructing the valvular tissue. In this surgery, a prosthetic ring is affixed to the mitral valve, by first suturing around the annulus of the valve and then implanting the ring of a chosen size, through the sutures onto the annulus [3]. Mitral valve repair is increasingly performed in a minimally invasive manner [4], with a reliance on image guidance, in particular endoscopic video for the reconstruction process.

Besides, the use of surgical simulators are becoming more popular in training and familiarizing the surgeons with the demanding surgical techniques. In our previous work, we showed how to simulate endoscopic surgeries on a patient-individual basis with the help of flexible 3*D*-printed mitral valve replica [10, 11]. The endoscopic data stream obtained during surgery or such simulations can be analysed in real time or retrospectively to extract quantitative information with regard to patient-valve geometry [21] or context-aware visualisations.

In particular, suture detection is one such task that can provide quantitative information. This information can then be used to analyse the suturing patterns, examine the correlation with different levels of expertise, understand the optimal suture configuration in the context of ring implantation, and to use the suture locations to create augmented reality visualisations [8]. The task of suture detection entails detecting the entry and exit point of the sutures around the annulus. More precisely, given an image and the corresponding suture locations for this image, the task is to predict the suture locations for unseen endoscopic images. There have been multiple approaches from the literature in the field of landmark detection, more commonly in facial landmark detection [26], pose estimation [2, 16] and medical landmark detection [23, 28] to tackle this problem. However, there are two important distinctions due to which these approaches are not directly applicable to our task. Firstly, for any given image, there exists a variable number of sutures, unlike a fixed number of facial or anatomical landmarks. Secondly, the points have a semantic meaning and are of single instance. This renders fixed regression-based approaches ill-suited to our task. Additionally, commonly used patch-based refinement of anatomical landmarks are inapt in this scenario.

In this approach, we seek to solve a multi-instance detection problem for 2D points. The approach is based on a heatmap which typically models the distribution of likelihood around the point as a Gaussian. In this paper, we extend on our previous conference work [24], where we proposed a simple U-net with a single channel output. The output map was thresholded and the centre of mass was calculated for each region to determine the final position of points.

In the work at hand, we introduce the usage of a differentiable *Gaussian* filter and a *Soft-Argmax* layer to enforce both, learning of the heatmap and further extracting the points from the heatmap, in a differentiable manner. We demonstrate an improvement compared to our previous baseline, and additionally perform experiments comparing various final layer configurations and loss functions. The approach is evaluated on two different domains, i.e. video data from simulated surgery and from real procedures are used. The data is made publicly available within the scope of the MICCAI AdaptOR2021 Challenge https://adaptor2021.github.io/. To be consistent with the challenge, we used the same data set split like in the challenge, which is slightly different from [24].

## Related work

### Regression-based approaches

Discriminative approaches to landmark detection comprise of regression or heatmap based methods. Regression-based methods directly estimate the landmark coordinates from the image. Duffner and Garcia [6] is one of the early works using neuronal layers to estimate facial feature positions. Subsequently, due to the exponential growth of deep learning tools and techniques, there have been a number of works that estimate this mapping with a neural network [26]. The regression-based methods model a mapping from the image space to the coordinate space, which is highly nonlinear and therefore difficult to learn. The approaches from the literature tackle this problem by using a *CNN* cascade or progressive refinement of the landmarks [13, 27].

In the field of medical imaging, landmark localization is a relevant step for tasks such as registration and augmented reality visualisations. Cascaded and stage-wise models [25, 28] are typically used for anatomical landmark regression. Sofka et al. [23] presented a landmark regressor for ultrasound image sequences that additionally imposes a temporal constraint with *LSTM* cells along with a centre-of-mass layer to extract landmark locations. However, all of the aforementioned methods predict a pre-defined number of landmarks, which allows to pre-determine the shape of the tensor to be regressed in the output layer of a network. Additionally, learning a transformation from the image space to the coordinate space is a highly nonlinear mapping which is further complicated by the variations in camera view, pose, illumination and scene composition.

### Heatmap-based approaches

Unlike regression-based approaches that directly regress on the coordinates, heatmaps model a distribution of likelihood around the points of interest. In the recent years, the field of landmark detection is moving towards the use of heatmap-based approaches [26], as they model a mapping from image-to-image space unlike regression models. A *Gaussian* distribution is commonly used to model the likelihood of the landmark locations. Typically, the heatmap approaches represent a single landmark in one channel, making it easy to perform differentiable operations or post-processing [5]. Bulat and Tzimiropoulos [2] proposed a two-stage network to regress on heatmaps and further finetune the landmarks in subsequent stages. The *Deep Alignment Network (DAN)* [17] processes the whole image in contrast to patches and finetunes the landmark estimates using heatmaps. In the medical domain, Zhang et al. [29] used a multi-task network to learn displacement maps using heatmaps. Payer et al. [18] proposed a two-stage heatmap-based network. All in all, similar to the regression approaches, the heatmap-based regression networks, model one heatmap per channel with a pre-defined set of landmarks.

In earlier works on a small intra-operative dataset [8, 9], we have used random forests and tailored post-processing for point detection and optical flow for point tracking. Our previous work on the same data base [24] formulates the landmark detection task as a deep learning-based approach, and demonstrates first results on intra-operative and surgical simulator datasets for heart surgeries. Hervella et al. [15] demonstrated a similar method for the case of retinal fundus images. However, the model has to learn from a heavily unbalanced dataset due to the nature of point landmarks in the context of a segmentation task. Brosch et al. [1] tackles this problem by using a novel objective function. In this paper, we extend our previous work [24] and tackle the unbalanced multi-instance sparse-segmentation task through the use of a differentiable convolutional *Soft-Argmax* layer combined with a balanced loss function. Iqbal et al. [16] used a differentiable *Soft-Argmax* layer to extract the landmark locations from the heatmap, but the problem formulation contained a single heatmap per channel for a pre-defined number of heatmaps. Chandran et al. [5] used a heatmap combined with the differentiable *Soft-Argmax* layer to extract regions of interest to provide a global context to landmark localization. In contrast to Iqbal et al. [16] and Chandran et al. [5], our approach uses a convolutional *Soft-Argmax* layer that is convolved spatially with the feature map, in order to impose stability in modeling a distribution and extracting multiple instances of landmarks from this distribution. Additionally, an $F_\beta$ loss is used to take into account the precision and recall for optimisation. Results compared to our previous baseline [24], along with ablations with various loss functions and output layer configurations, are presented.

## Methods

### Task formulation

**Fig. 1:**
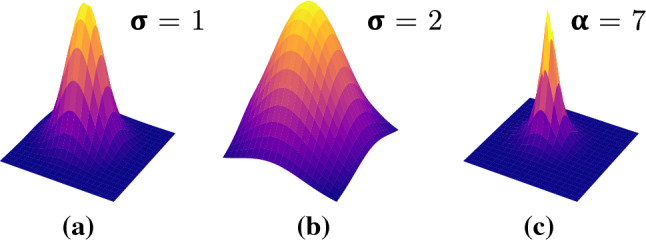
Distribution functions. **a** *Gaussian*, $\sigma =1$ **b** *Gaussian*, $\sigma =2$ **c** *Tanh*, $\alpha =7$

The labels $s_i \in S, i \in 1, 2 \ldots N$ can be equivalently represented as a binary mask, where $p_{(x_i, y_i)} \in \{0, 1\}, x_i \in 1, 2 \ldots ,H, y_i \in 1, 2 \ldots ,W$, denotes the pixel value at image location $(x_i, y_i)$, with a value of 1 in the suture locations and 0 otherwise. Alternatively, the position of each suture instance can be modelled by a distribution centred around the location that represents the likelihood of the pixel being a landmark location. In this case, $p_{(x_i, y_i)}$ takes values in [0, 1]. In this work, we present and compare two different distribution functions to model the heatmap, namely the *Gaussian* and the *Tanh* distribution. In the case of the *Gaussian* distribution, the spread is controlled by the variable $\sigma _1$, which we set to $\sigma _1=1$, 2, and 3. The *Tanh* distribution is a sharper distribution, where we set the variable $\alpha =3.5\times \sigma$ that controls the spread of the distribution. We experiment with the values of $\alpha =7$ and $\alpha =10.5$. An illustration comparing the *Gaussian* and *Tanh* distribution is provided in Fig. 1.

### Network architecture

The labels $\hat{y}_{j}$ for unseen endoscopic images can be estimated by a neural network $\phi (x_j, y_j, \theta )$ with parameters $\theta$. A U-Net-based [20] architecture is used, similar to the one described in our previous work [24], but using *ReLU* activations for the convolutional layers. *RGB* 3-channel images of size $288 \times 512$ are provided to the model as input. A mask of the same size is created from the labelled suture locations. A distribution function centred around each suture point is applied to the binary mask as described in Sect. 3.1.

**Fig. 2:**
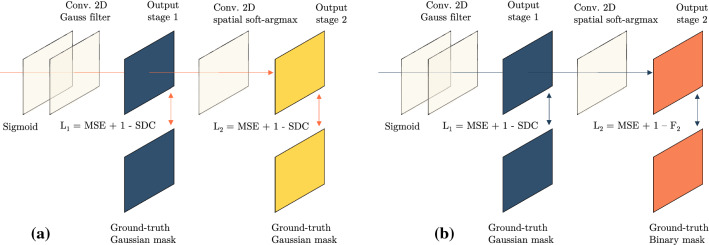
Overview of the variants for the proposed suture detection network. **a** Variant 1: A *Gaussian* mask is used at both output stages. **b** Variant 2: A binary mask is used at output Stage 2

Furthermore, a differentiable *Gaussian* filter with spread $\sigma _2$ is applied to the output of the *Sigmoid* layer. Different values of $\sigma _1$ and $\sigma _2$ are applied and a comparison is presented in Sect. 5.1. A similarity loss $\mathcal {L}_{1}$ between the filtered output (*Output Stage* 1 in Fig. 2a, b) and the ground-truth heatmap is applied that enforces the model to learn the likelihood distribution of the suture locations. The *Gaussian* filter encourages the model to learn a smooth distribution around the predicted locations. Additionally, the filtered output is fed through a differentiable convolutional 2D spatial *Soft-Argmax* layer to produce the final output of the model (*Output Stage* 2 in Fig. 2a, b). In this layer, a *Soft-Argmax* kernel of size $(3 \times 3)$, with a stride of 1 and a padding of 1 is convolved with the output from the previous layer. The layer is implemented using the *Kornia* [19] library. An additional similarity loss $\mathcal {L}_{2}$ is applied at this stage, as shown in Fig. 2a and b. Other works [5, 16] demonstrated the use of a differentiable *Soft-Argmax* layer in extracting the landmarks from the heatmaps, where a single landmark is used per channel of the heatmap, and as a result, the *Soft-Argmax* layer yields the landmark locations. In contrast, we represent all suture locations in a single channel. Therefore, the convolutional *Soft-Argmax* layer functions as a local non-maximum suppression for the points with low likelihood of being a suture point.

At the output of stage 1, a loss function of the form $\mathcal {L}_{1} = \mathrm{MSE} + 1 - \mathrm{SDC}$ is applied between the predicted suture points and the ground-truth heatmap, where MSE is the *Mean Squared Error* and SDC is the *S*ø*rensen Dice Coefficient*. For the model variant 1 at the Output Stage 2 as shown in Fig. 2a, the same loss $\mathcal {L}_{2} = \mathrm{MSE} + 1 - \mathrm{SDC}$ is applied for a heatmap ground-truth. For the model variant 2 at the Output Stage 2 as shown in Fig. 2b, the output is optimised with a binary ground-truth mask. In this case, the true and false pixel classes are even more unbalanced. An asymmetric similarity loss function that can weigh the precision and recall is previously shown to perform better for unbalanced classes [14], in comparison with the dice coefficient. We therefore apply a balanced $F_\beta$ loss function for the predicted and ground-truth binary masks

1 $$\begin{aligned} F_\beta = \frac{{(1+\beta ^2)}{\sum _{i=1}^{N} {p_i}{g_i}}}{{{(1+\beta ^2)}{\sum _{i=1}^{N} {p_i}{g_i}}} +{{\beta ^2}{\sum _{i=1}^{N} {(1-p_i)}{g_i}}} + {{\sum _{i=1}^{N} {p_i}{(1-g_i)}}}} \end{aligned}$$

where $p_i$ is the likelihood of a pixel being a suture, and the binary label $g_i \in \{0,1\}$ denotes the presence of a suture point. A value of $\beta =2$ is used, to penalise the number of false negatives. The final suture detection model, jointly optimises the loss functions $\mathcal {L}_{1}$ and $\mathcal {L}_{2}$, given by

2 $$\begin{aligned} \mathcal {L}_{\phi (x_j, y_j, \theta )} = \min _\theta (\mathcal {L}_{1} + \mathcal {L}_{2}). \end{aligned}$$

The predicted heatmaps that are obtained after evaluation are thresholded with $t=0.5$, and the centre-of-mass of the local clusters are computed, to extract the predicted suture coordinates, that are then evaluated with the labelled suture coordinates.

### Evaluation

**Fig. 3:**
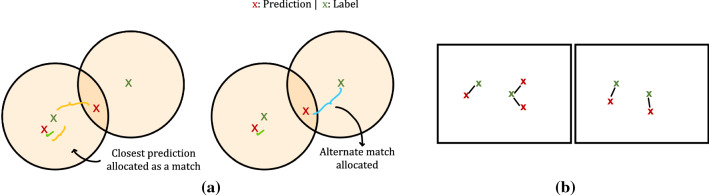
**a** An illustration of allocating matches between the predicted and ground-truth suture labels. **b** A comparison of cases that yield a similar RMSE metric

A suture point is considered to be successfully predicted, if the distance between the predicted and ground-truth point is less than 6 pixels, as proposed in [24]. If multiple points are matched with the ground-truth, then the point closest to the ground-truth is chosen as the predicted point. Once the closest match is allocated the second-best match is used to match other labels in the image. An illustration of this is shown in Fig. 3a. From these predicted coordinates, the number of *True Positives (TP)*, *False Positives (FP)*, and *False Negatives (FN)* is determined. Furthermore, the *Positive Predicted Value (PPV)* is computed as $\mathrm{PPV} = \mathrm{TP}/(\mathrm{TP} + \mathrm{FP})$ and the *True Positive Rate (TPR)* is determined as $\mathrm{TPR} = \mathrm{TP}/(\mathrm{TP} + \mathrm{FN})$. In order to compare the performance of two different models, the $F_1$ score is computed by taking the harmonic mean of PPV and TPR as $F_1 = (\mathrm{PPV} \times \mathrm{TPR}) / (\mathrm{PPV} + \mathrm{TPR})$. Additionally in this work, we compute the root mean square error of the Euclidean distance as a localisation metric. For each predicted point in an image, the Euclidean distances to all ground-truth points are computed and the least distance is chosen. This is then averaged across all predicted points in an image, for the images that have predictions. For images without predicted points, the metric cannot be computed, and we therefore additionally report the number of images where this occurs in Table 3. For RMSE computation, we consider each predicted point in the calculation, i.e. each point has a match. Points which are further away without a match would otherwise not be penalized in the metric.

## Data and experiments

### Datasets

In this work, datasets from two domains are used for the experiments, namely the intra-operative and the surgical simulator domain. The data in the intra-operative domain comes from endoscopic frames captured during mitral valve repair surgery. The intra-operative data forms a heterogeneous dataset comprising of frames with widely varying camera view, scale, lighting sources, and white balance. Additionally, the dataset also contains the presence of endoscopic artefacts caused due to occlusion and specular reflections. The intra-operative datasets are split on a surgery level, like in the mentioned challenge. Firstly, a dataset for cross-validation (*A*.1) is created, comprising of four surgeries, with a total of 2376 images. It is important to note that the validation set is not used for fine-tuning the model performance, for example, using early stopping. Therefore, the validation set functions as an unseen dataset for the model. Additionally, a second independent intra-operative test set is created (*A*.2). The data in the surgical simulator domain comes from endoscopic capture of the surgical training and planning sessions on the mitral valve silicone replica models [10]. A simulator dataset (*B*.1) is created from ten such simulator sessions, with a total of 2708 images.

The endoscopic videos are captured in a top-down stereo format, after which relevant frames are extracted. The left and right images of the stereo pair are treated independently. The suture points identified in these frames are then manually labelled using the annotation tool *labelme*. For further details about the endoscopic capture of the data, the reader is referred to our previous work [22, 24]. For this work, the previously annotated suture point labels are revisited and quality-checked. After correction, the intra-operative cross-validation dataset contains a total of 23, 938 sutures, and the simulator dataset a total of 33, 872 sutures. The data is released within the scope of the AdaptOR2021 MICCAI challenge at https://adaptor2021.github.io/

The endoscopic frames are resized to a resolution of $288 \times 512$, and image and mask augmentations are applied, before feeding to the model. Similar to our previous work [24], the dataset was augmented with vertical and horizontal flip, rotation of $\pm 60^{\circ }$, and affine translations with $\pm 10\%$. Additionally, image augmentations comprising of pixel shifting in range $\pm 1\%$, shearing in range $\pm 0.1$, brightness in range $\pm 0.2$, contrast in range from 0.3 to 0.5, random saturation in range from 0.5 to 2.0, and hue in range of $\pm 0.1$ are applied. All augmentations are applied with a probability of $50\%$.

### Experiments

Firstly, in the intra-operative domain, a fourfold cross-validation is performed on dataset *A*.1. Additionally, the models are evaluated on an independent test set *A*.2. In the simulator domain, a fivefold cross-validation is performed on the dataset *B*.1. Our baseline results were presented in [24] for a *Gaussian* heatmap with a $\sigma _1 = 1$. Here, we recompute the baseline for $\sigma _1\in {1, 2, 3}$, with the refined labels and the data split as described in Sect. 4.1. We present a comparison of the model variants described in Sect. 3.2 with the baseline. Furthermore, we perform a sensitivity analysis with different parameters of the heatmap distribution, namely *Gaussian* with $\sigma _1\in {1, 2, 3, 4}$, $\sigma _2\in {1, 2, 3}$ and *Tanh* with $\alpha \in {7, 10.5}$. Here, the effect on the model performance in relation to varying $\sigma _1$ and $\sigma _2$ are presented. Moreover, we present an evaluation with a localisation metric, as described in Sect. 3.3. Finally, we present a comparison of the evaluation with 3 different radii around the ground-truth point, namely 6, 8, and 10 pixels, for the best-performing model in each domain. The network is trained with a learning rate of 0.001, with an *Adam* optimizer. A learning rate decay scheme is used to reduce the rate by a factor of 0.1 upon a plateau for 10 epochs. The models were trained on one of *NVIDIA Quattro P6000*, *NVIDIA TITAN RTX*, or *NVIDIA TITAN V*. The *PyTorch* library was used to implement the model pipeline.

## Results and discussion

### Results

The results of the fourfold cross-validation in the intra-operative domain on dataset *A*.1 are presented in Table 1(a). Additionally, an evaluation of two variants of the proposed model in comparison with the baseline from our previous work [24], on the intra-operative test set *A*.2 is shown in Table 1(b). In the simulator domain, results of the fivefold cross-validation are presented in Table 1 (c). Samples from prediction from cross-validation in the intra-operative (*A*.1) and the simulator domain (*B*.1) are shown in Fig. 5.

Firstly, from the cross-validation in the intra-operative dataset (*A*.1), it can be seen that in comparison with the our previous baseline with the value of $\sigma _1=1$ [24], the performance of the model increases with $\sigma _1=2$. For both the values of $\sigma _1=2, \sigma _1=3$, the model performs better than while using a *Tanh* distribution, with respective values of $\alpha =7, \alpha =10.5$ (mean $F_1$ $+0.0082$ for $\sigma _1=3, \alpha =10.5$ OR *A*.1 c.f. Table 1a). To recall, $\sigma _1$ here denotes the spread of the *Gaussian* distribution used to create the masks. $\sigma _2$ refers to the parameters of the local differentiable *Gaussian* layer used in the proposed model variants.

In the intra-operative dataset, the Variant 1 of the proposed model with $\sigma _1=3$ outperforms the baseline from our previous work [24] with $\sigma _1=1$ (mean $F_1$ $+0.0082$ for $\sigma _1=3$ OR *A*.1 c.f. Table 1a). Variant 1 also outperforms the baseline model with the same $\sigma _1$ value of 3 (mean $F_1$ $+0.0080$ for $\sigma _1=3$ OR *A*.1 c.f. Table 1 (a)). In this case, the difference is the differentiable local *Gaussian* and *SoftArgMax* layers in the model architecture. A larger spread of the *Gaussian* distribution provides more likelihood values around every landmark and additionally reduces the imbalance of the pixels in the dataset, thereby helping the model learn better. However, a larger spread around the suture point also means that the model is prone to confounding from nearby points due to overlapping distributions. Similarly, in the Simulator domain, the proposed model Variant 1 with $\sigma _1=2$ outperforms the baseline from our previous work [24] (mean $F_1$ $+0.0865$ Sim *B*.1 c.f. Table 1 (c)), with $\sigma =1$, and the baseline model with the same value of $\sigma _1=2$ (mean $F_1$ $+0.0354$ Sim *B*.1 c.f. Table 1 (c)).

**Table 1:** Results of baselines and model variants on (a) OR Cross-validation dataset *A*.1, (b) OR Test dataset *A*.2, (c) Sim Cross-validation dataset *B*.1. Best $F_1$ scores are highlighted in bold

Experiment	Mask distribution	PPV	TPR	$F_1$
(a) *Cross-validation on* *OR* *data* (*A*.1) *(Higher is better)*				
Baseline [24]	Gauss, $\sigma =1$	$62.2700 \pm 9.54$	$33.1350 \pm 6.68$	$0.4376 \pm 0.06$
Baseline [24]	Gauss, $\sigma =2$	$64.4450 \pm 11.01$	$37.6525 \pm 8.46$	$0.4720 \pm 0.05$
Baseline [24]	Gauss, $\sigma =3$	$65.8775 \pm 8.95$	$37.8200 \pm 10.58$	$0.4718 \pm 0.08$
Baseline [24]	Tanh, $\alpha =7$	$69.2850 \pm 6.42$	$34.6900 \pm 5.74$	$0.4494 \pm 0.05$
Baseline [24]	Tanh, $\alpha =10.5$	$68.3550 \pm 7.90$	$35.2900 \pm 5.55$	$0.4636 \pm 0.06$
Variant 1	Gauss, $\sigma _1 =2$	$68.8400 \pm 7.84$	$38.0650 \pm 9.30$	$0.4789 \pm 0.06$
Variant 1	Gauss, $\sigma _1 =3$	$67.2100 \pm 11.08$	$39.5225 \pm 10.23$	$\mathbf {0.4798 \pm 0.04}$
Variant 1	Gauss, $\sigma _1 =4$	$67.3275 \pm 16.92$	$29.3600 \pm 7.77$	$0.3711 \pm 0.04$
Variant 2	Gauss, $\sigma _1 =2$	$74.7625 \pm 8.30$	$33.9950 \pm 9.99$	$0.4576 \pm 0.09$

Experiment	Mask distribution	PPV	TPR	$F_1$
(b) *Results on additional* *OR* *data test set* (*A*.2)				
Baseline [24]	Gauss, $\sigma =2$	$76.0150 \pm 7.36$	$28.5025 \pm 2.53$	$0.4126 \pm 0.02$
Baseline [24]	Gauss, $\sigma =3$	$72.6550 \pm 1.43$	$28.9350 \pm 1.14$	$0.4138 \pm 0.01$
Baseline [24]	Tanh, $\alpha =7$	$76.2525 \pm 3.52$	$26.3000 \pm 2.20$	$0.3902 \pm 0.02$
Baseline [24]	Tanh, $\alpha =10.5$	$74.1375 \pm 5.83$	$28.2450 \pm 2.18$	$0.4084 \pm 0.03$
Variant 1	Gauss, $\sigma _1 =2$	$76.6475 \pm 5.31$	$28.0750 \pm 2.92$	$0.4086 \pm 0.02$
Variant 1	Gauss, $\sigma _1 =3$	$73.3200 \pm 2.94$	$30.4550 \pm 3.76$	$\mathbf {0.4290 \pm 0.04}$
Variant 1	Gauss, $\sigma _1 =4$	$67.5825 \pm 8.42$	$22.3425 \pm 3.05$	$0.3317 \pm 0.03$
Variant 2	Gauss, $\sigma _1 =2$	$78.3350 \pm 2.88$	$26.3875 \pm 1.79$	$0.3945 \pm 0.02$

Experiment	Mask distribution	PPV	TPR	$F_1$
*(c) Cross-validation on Simulator data* (*B*.1)				
Baseline [24]	Gauss, $\sigma =1$	$78.3260 \pm 4.44$	$61.6360 \pm 7.96$	$0.6869 \pm 0.06$
Baseline [24]	Gauss, $\sigma =2$	$81.3900 \pm 5.18$	$67.9540 \pm 9.82$	$0.7380 \pm 0.08$
Variant 1	Gauss, $\sigma _1 =2$	$83.2840 \pm 3.76$	$72.4860 \pm 8.56$	$\mathbf {0.7734 \pm 0.06}$
Variant 1	Gauss, $\sigma _1 =3$	$78.4560 \pm 6.38$	$64.6160 \pm 9.72$	$0.7057 \pm 0.07$
Variant 2	Gauss, $\sigma _1 =2$	$81.0100 \pm 5.92$	$73.7160 \pm 7.34$	$0.7704 \pm 0.06$

In the intra-operative domain, Variant 2 of the proposed model does not outperform the baseline with the corresponding $\sigma _1$ value (mean $F_1$ $-0.0144$ for $\sigma _1=3$ OR *A*.1 c.f. Table 1 (a)). In the simulator domain however, the Variant 2 outperforms the corresponding baseline (mean $F_1$ $+0.0324$ Sim *B*.1 c.f. Table 1 (c)). Binary masks in this case, without a likelihood distribution, constitute a highly imbalanced dataset, which hampers the learning process and affects performance. In both domains, the model Variant 1 yields the best performing model.

**Fig. 4:**
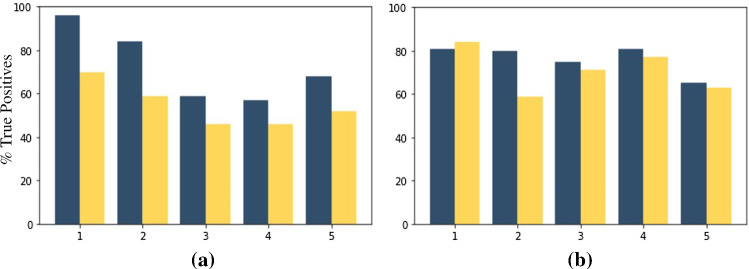
A comparison of the percentage of True Positives detected in each fold in the simulator domain cross-validation dataset *B*.1. Blue bars denote the model with $\sigma _1=2$, $\sigma _2=1$; Yellow bars denote the model with with $\sigma _1=3$, $\sigma _2=1$; **a** provides a comparison of the subset containing points close to each other. **b** Subset not close to each other

Furthermore, for values $\sigma _1 = 2$, $\sigma _1 = 3$, the values of $\sigma _2$ are varied between 1, 2, and 3 and the results are presented in Table 2. In each domain, the best performing model is with the value $\sigma _2 = 1$. In both the cases of intra-operative and the simulator domains, there is a best-performing value of ($\sigma _1$, $\sigma _2$) after which the performance of the model drops. In the case of the intra-operative domain, this performance occurs at ($\sigma _1=3$, $\sigma _2=1$) and in the case of the simulator domain, at ($\sigma _1=2$, $\sigma _2=1$). This is due to the trade-off that occurs while increasing the spread of the distribution around the suture points. In order to understand this trade-off, the model performance is analysed at the level of two different subsets. Firstly, a subset of close-points are defined as the points that are within a distance of 15 pixels within each other. The rest of the points are categorised as non-close points. Then, the change in the *True Positive* points, as we go from $\sigma _1=2$ to $\sigma _1=3$ is analysed. An example illustration in the simulator domain is shown in Fig. 4. It can be seen that the drop in the percentage of *True Positives* is higher in the case of the close subset in comparison with the points that are not located close to each other.

**Table 2:** Comparison of different *Gaussian* values used for creating the suture masks ($\sigma _1$) versus the *Gaussian* values used in the local differentiable *Gaussian* layer ($\sigma _2$); on the OR (*A*.1) and simulator dataset (*B*.1). Highest values for each metric are highlighted in bold

Metric	Experiment	Mask distribution	$\sigma _2=1$	$\sigma _2=2$	$\sigma _2=3$
*(a) Cross-validation on OR dataset* (*A*.1) *(Higher is better)*					
PPV	Variant 1	Gauss, $\sigma _1 =2$	$68.8400 \pm 7.84$	$\mathbf {75.1825 \pm 6.42}$	$68.3875 \pm 6.90$
	Variant 1	Gauss, $\sigma _1 =3$	$67.2100 \pm 11.08$	$70.6550 \pm 13.90$	$63.4375 \pm 13.53$
TPR	Variant 1	Gauss, $\sigma _1 =2$	$38.0650 \pm 9.306$	$35.4575 \pm 6.26$	$35.5925 \pm 9.03$
	Variant 1	Gauss, $\sigma _1 =3$	$\mathbf {39.5225 \pm 10.23}$	$33.9025 \pm 3.74$	$35.8525 \pm 8.46$
$F_1$	Variant 1	Gauss, $\sigma _1 =2$	$0.4789 \pm 0.06$	$0.4781 \pm 0.06$	$0.4582 \pm 0.07$
	Variant 1	Gauss, $\sigma _1 =3$	$\mathbf {0.4798 \pm 0.04}$	$0.4503 \pm 0.03$	$0.4485 \pm 0.07$

Metric	Experiment	Mask distribution	$\sigma _2=1$	$\sigma _2=2$	$\sigma _2=3$
*(b) Cross-validation on Simulator dataset* (*B*.1)					
PPV	Variant 1	Gauss, $\sigma _1 =2$	$\mathbf {83.2840 \pm 3.76}$	$80.8760 \pm 7.53$	$82.0220 \pm 5.14$
	Variant 1	Gauss, $\sigma _1 =3$	$78.4560 \pm 6.38$	$77.8520 \pm 9.46$	$79.6720 \pm 4.77$
TPR	Variant 1	Gauss, $\sigma _1 =2$	$\mathbf {72.4860 \pm 8.56}$	$68.0740 \pm 5.00$	$64.9800 \pm 9.81$
	Variant 1	Gauss, $\sigma _1 =3$	$64.6160 \pm 9.72$	$66.1020 \pm 11.25$	$67.2180 \pm 10.76$
$F_1$	Variant 1	Gauss, $\sigma _1 =2$	$\mathbf {0.7734 \pm 0.06}$	$0.7368 \pm 0.05$	$0.7188 \pm 0.06$
	Variant 1	Gauss, $\sigma _1 =3$	$0.7057 \pm 0.07$	$0.7116 \pm 0.09$	$0.7254 \pm 0.07$

Moreover, we compute the root-mean-square error of the Euclidean distance as explained in Sect. 3.3, the results of which are presented in Table 3. As given in Table 3, the results are different as compared to the $F_1$ score metric presented in Table 2. Although the RMSE distance provides an indication of the closeness of the points to the ground truth labels, it is difficult to analyse a case where the RMSE of two models are the same despite one of the models predicting more False Positives, since the metric is averaged over each predicted point. An example of this is shown in Fig. 3b. Finally, we present an evaluation with three different radii around the ground-truth point for which a match is allocated, namely six pixels, eight pixels, and ten pixels, for the best-performing model in each domain, as given in Table 4.

**Table 3:** Comparison of the RMSE distance with different *Gaussian* values used for creating the suture masks ($\sigma _1$) versus the *Gaussian* values used in the local differentiable *Gaussian* layer ($\sigma _2$); on the (a) OR cross-validation dataset (*A*.1) (b) additional OR test dataset (*A*.2), and the simulator cross-validation dataset (*B*.1). Lowest RMSE values are highlighted in bold

*(a) Cross-validation on OR dataset* (*A*.1) *(Lower is better)*				
(Computed for 2187 out of 2376 images)				
Experiment	Mask distribution	$\sigma _2=1$	$\sigma _2=2$	$\sigma _2=3$
Variant 1	Gauss, $\sigma _1 =2$	$22.99 \pm 6.61$	$\mathbf {17.20 \pm 4.26}$	$28.84 \pm 11.12$
Variant 1	Gauss, $\sigma _1 =3$	$25.01 \pm 12.84$	$24.82 \pm 17.65$	$28.55 \pm 13.96$

*(b) Results on additional* *OR* *data test set* (*A*.2)				
(Computed for 391 out of 500 images)				
Experiment	Mask distribution	$\sigma _2=1$	$\sigma _2=2$	$\sigma _2=3$
Variant 1	Gauss, $\sigma _1 =2$	$17.2489 \pm 3.00$	$17.3576 \pm 4.65$	$17.4979 \pm 2.01$
Variant 1	Gauss, $\sigma _1 =3$	$19.6230 \pm 4.25$	$\mathbf {17.1994 \pm 3.67}$	$24.5889 \pm 2.73$

*(c) Cross-validation on simulator data* (*B*.1)				
(Computed for 2678 out of 2708 images)				
Experiment	Mask distribution	$\sigma _2=1$	$\sigma _2=2$	$\sigma _2=3$
Variant 1	Gauss, $\sigma _1 =2$	$\mathbf {11.2789 \pm 6.00}$	$13.1211 \pm 10.95$	$11.4301 \pm 6.34$
Variant 1	Gauss, $\sigma _1 =3$	$13.4286 \pm 8.49$	$14.1760 \pm 10.45$	$13.8515 \pm 6.70$

**Table 4:** Comparison of evaluation with three different radii around the ground-truth point, for the best performing models on (a) OR cross-validation dataset *A*.1, (b) additional OR Test dataset *A*.2, (c) Simulator cross-validation dataset *B*.1. Highest values for each metric are highlighted in bold

Experiment	Mask distribution	Radius	PPV	TPR	$F_1$
*(a) Cross-validation on* $\textit{OR}$ *data* (*A*.1) *(Higher is better)*					
Variant 1	Gauss, $\sigma _1 =3$	6px	$67.2100 \pm 11.0821$	$39.5225 \pm 10.2259$	$0.4798 \pm 0.0427$
Variant 1	Gauss, $\sigma _1 =3$	8px	$69.1575 \pm 10.80$	$39.5225 \pm 10.23$	$0.4946 \pm 0.05$
Variant 1	Gauss, $\sigma _1 =3$	10px	$\mathbf {70.0500 \pm 10.59}$	$\mathbf {41.3800 \pm 11.14}$	$\mathbf {0.5014 \pm 0.05}$

Experiment	Mask distribution	Radius	PPV	TPR	$F_1$
*(b) Results on additional* *OR* *data test set* (*A*.2)					
Variant 1	Gauss, $\sigma _1 =3$	6px	$73.3200 \pm 2.94$	$30.4550 \pm 3.76$	$0.4290 \pm 0.04$
Variant 1	Gauss, $\sigma _1 =3$	8px	$75.7525 \pm 2.68$	$30.4550 \pm 3.76$	$0.4433 \pm 0.04$
Variant 1	Gauss, $\sigma _1 =3$	10px	$\mathbf {76.4100 \pm 2.86}$	$\mathbf {31.74 \pm 3.91}$	$\mathbf {0.4471 \pm 0.04}$

Experiment	Mask distribution	Radius.	PPV	TPR	$F_1$
*(c) Cross-validation on simulator data* (*B*.1)					
Variant 1	Gauss, $\sigma _1 =2$	6px	$83.2840 \pm 3.76$	$72.4860 \pm 8.56$	$0.7734 \pm 0.06$
Variant 1	Gauss, $\sigma _1 =2$	8px	$84.0475 \pm 3.95$	$69.9425 \pm 7.70$	$0.7689 \pm 0.06$
Variant 1	Gauss, $\sigma _1 =2$	10px	$\mathbf {85.1800 \pm 4.06}$	$\mathbf {72.0200 \pm 7.46}$	$\mathbf {0.7790 \pm 0.05}$

### Discussion

**Fig. 5:**
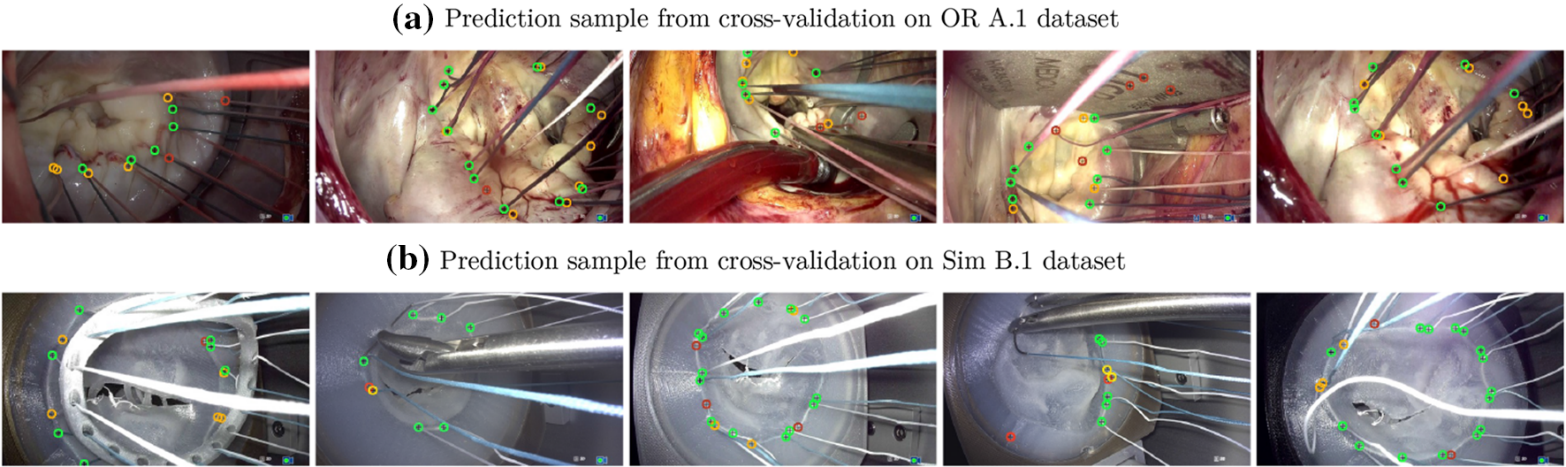
Samples of prediction from **a** Cross-validation on the OR dataset *A*.1 **b** Cross-validation on the Sim dataset *B*.1. Green—True Positive, Orange—False Negative, Red—False Positive

In this paper, as an extension to our previous work [24], we tackle the suture detection task by introducing a differentiable 2D *Gaussian* filter layer, and an additional differentiable convolutional 2D spatial convolutional *Soft-Argmax* layer. Unlike other works [5, 16] that use a *Soft-Argmax* layer to directly extract the landmarks from the heatmap from a single channel, we present its use as a form of local non-maximum suppression to filter out points with low likelihood of being a suture. Firstly, we perform experiments comparing the baseline from our previous work [24], with different values of $\sigma _1$. Here, we also present comparison of the *Gaussian* distribution with a *Tanh* distribution with a similar spread. Then, we present two variants of our proposed model in comparison with the baseline (c.f. Table 1). Further, we present experiments by varying values of $\sigma _1\in 1, 2, 3, 4$ and $\sigma _2\in 1, 2, 3$ (c.f. Table 2). In addition to the evaluation with the $F_1$ score, we compute an RMSE metric (c.f. Table 3). The RMSE metric has a limitation by comparing the models while taking into account the False Positives, as explained in Sect. 3.3. In the intra-operative domain, the Variant 1 with values ($\sigma _1=3, \sigma _2=1$) is the best performing model with an $F_1$ score of $0.4798\pm 0.04$ OR *A*.1, $0.4290\pm 0.04$ OR *A*.2 c.f. Table 1(a) and (b), and Variant 1 with values ($\sigma _1=2, \sigma _2=1$) is the best performing model with an $F_1$ score of $0.7734\pm 0.06$ Simulator *B*.1, c.f. Table 1 (c). The intra-operative dataset is a highly heterogeneous dataset comprising of images from different viewing angles, scale, light sources, and white balance. Furthermore, the intra-operative datasets contain endoscopic artefacts caused due to specularities, and occlusions from tissue or surgical instruments in the scene which make it a challenging dataset to learn from. Finally, it is often the case that two sutures are stitched close to each other. This makes it further difficult for the model, and a human reader, to distinguish nearby sutures. In particular, the final 2D *Gaussian* filter layer and the convolutional 2D spatial *Soft-Argmax* layer operate locally with a window and are prone to be confounded by closely occurring suture points. This is especially true in the case of higher *Gaussian* $\sigma _1$ values, as can be seen in Table 2. Varying the values $\sigma _1$ and $\sigma _2$ each have an effect on model performance in relation to the number of points in the dataset that are nearby or farther away from each other. In this regard, an adaptive variation in the *Gaussian* distribution is a potential future work, to handle these variations (Fig. 5).

Besides providing quantitative information for analysis of endoscopic data, the learned representations from the suture detection task can also be used to support other learning objectives. In particular, this task is relevant in the context of generative models to transform data from the simulator to the intra-operative domain [7, 12]. In our recent work [22], we show that suture detection models can be used to mutually improve generative domain transformation in endoscopy.

## Conclusion

In this work, we tackle the task of suture detection for endoscopic images by formulating it as a multi-instance sparse heatmap-regression problem. We extend our previous work [24] and improve upon the previously reported baselines. We introduce a novel *Gaussian* filter layer and a differentiable convolutional *Soft-Argmax* layers. We compare multiple distribution functions and present two variants of the model that outperform the baselines. Suture detection is an important task that can further be used towards supporting the learning of related objectives for endoscopic image analysis.

## Data Availability

The data is released within the scope of the AdaptOR2021 MICCAI challenge at https://adaptor2021.github.io/.

## Declarations

### Code availability

The authors plan to make the code publicly available in the subsequent months.

## References

[1] Brosch T, Yoo Y, Tang LYW, Li DKB, Traboulsee A, Tam R (2015) Deep convolutional encoder networks for multiple sclerosis lesion segmentation. In: Navab N, Hornegger J, Wells WM, Frangi AF (eds) Medical image computing and computer-assisted intervention—MICCAI 2015, lecture notes in computer science, pp 3–11. Springer, Cham. 10.1007/978-3-319-24574-4_1

[2] Bulat A, Tzimiropoulos G (2016) Human pose estimation via convolutional part heatmap regression. In: Leibe B, Matas J, Sebe N, Welling M (eds) Computer vision—ECCV 2016, lecture notes in computer science, pp 717–732. Springer, Cham. 10.1007/978-3-319-46478-7_44

[3] Carpentier A, Deloche A, Dauptain J, Soyer R, Blondeau P, Piwnica A, Dubost C, McGoon DC (1971). A new reconstructive operation for correction of mitral and tricuspid insufficiency. J Thorac Cardiovasc Surg.

[4] Casselman FP, Van Slycke S, Wellens F, De Geest R, Degrieck I, Van Praet F, Vermeulen Y, Vanermen H (2003) Mitral valve surgery can now routinely be performed endoscopically. Circulation, 108(10_suppl_1):II–48. 10.1161/01.cir.0000087391.49121.ce 10.1161/01.cir.0000087391.49121.ce 12970208

[5] Chandran P, Bradley D, Gross M, Beeler T (2020) Attention-driven cropping for very high resolution facial landmark detection. In: 2020 IEEE/CVF conference on computer vision and pattern recognition (CVPR), pp 5860–5869. 10.1109/CVPR42600.2020.00590

[6] Duffner S, Garcia C (2005) A connexionist approach for robust and precise facial feature detection in complex scenes. In: ISPA 2005. Proceedings of the 4th international symposium on image and signal processing and analysis, 2005, pp 316–321. 10.1109/ISPA.2005.195430. ISSN: 1845-5921

[7] Engelhardt S, De Simone R, Full PM, Karck M, Wolf I (2018) Improving surgical training phantoms by hyperrealism: deep unpaired image-to-image translation from real surgeries. In: MICCAI, pp 747–755. Springer

[8] Engelhardt S, De Simone R, Zimmermann N, Al-Maisary S, Nabers D, Karck M, Meinzer HP, Wolf I (2014) Augmented reality-enhanced endoscopic images for annuloplasty ring sizing. In: Augmented environments for computer-assisted interventions, pp 128–137. Springer

[9] Engelhardt S, Kolb S, De Simone R, Karck M, Meinzer HP, Wolf I (2016) Endoscopic feature tracking for augmented-reality assisted prosthesis selection in mitral valve repair. In: Proceedings of the SPIE, medical imaging: image-guided procedures, robotic interventions, and modeling, vol 9786, pp 402–408

[10] Engelhardt S, Sauerzapf S, Brčić A, Karck M, Wolf I, De Simone R (2019). Replicated mitral valve models from real patients offer training opportunities for minimally invasive mitral valve repair. Interact Cardiovasc Thorac Surg.

[11] Engelhardt S, Sauerzapf S, Preim B, Karck M, Wolf I, De Simone R (2019). Flexible and comprehensive patient-specific mitral valve silicone models with chordae tendinae made from 3D-printable molds. Int J Comput Assist Radiol Surg.

[12] Engelhardt S, Sharan L, Karck M, De Simone R, Wolf I (2019) Cross-Domain conditional generative adversarial networks for stereoscopic hyperrealism in surgical training. In: MICCAI. 10.1007/978-3-030-32254-0_18

[13] Fan H, Zhou E (2016). Approaching human level facial landmark localization by deep learning. Image Vis Comput.

[14] Hashemi SR, Salehi SSM, Erdogmus D, Prabhu SP, Warfield SK, Gholipour A (2019) Asymmetric loss functions and deep densely connected networks for highly imbalanced medical image segmentation: application to multiple sclerosis lesion detection. IEEE Access?: practical innovations, open solutions 7:721–1735. 10.1109/ACCESS.2018.2886371 10.1109/ACCESS.2018.2886371 PMC6746414 31528523

[15] Hervella ÁS, Rouco J, Novo J, Penedo MG, Ortega M (2020) Deep multi-instance heatmap regression for the detection of retinal vessel crossings and bifurcations in eye fundus images. Comput Methods Programs Biomed 186:105201. 10.1016/j.cmpb.2019.105201 10.1016/j.cmpb.2019.105201 31783244

[16] Iqbal U, Molchanov P, Breuel T, Gall J, Kautz J (2018) Hand pose estimation via latent 2.5D heatmap regression. In: ECCV. 10.1007/978-3-030-01252-6_8

[17] Kowalski M, Naruniec J, Trzcinski T (2017) Deep Alignment Network: A Convolutional Neural Network for Robust Face Alignment. In: 2017 IEEE Conference on Computer Vision and Pattern Recognition Workshops (CVPRW), pp. 2034–2043. 10.1109/CVPRW.2017.254. ISSN: 2160-7516

[18] Payer C, Štern D, Bischof H, Urschler M (2019). Integrating spatial configuration into heatmap regression based CNNs for landmark localization. Med Image Anal.

[19] Riba E, Mishkin D, Ponsa D, Rublee E, Bradski G (2020) Kornia: an open source differentiable computer vision library for pytorch. In: Winter conference on applications of computer vision

[20] Ronneberger O, Fischer P, Brox T (2015) U-Net: convolutional networks for biomedical image segmentation. In: Navab N, Hornegger J, Wells WM, Frangi AF (eds) Medical image computing and computer-assisted intervention—MICCAI 2015, lecture notes in computer science, pp 234–241. Springer, Cham. 10.1007/978-3-319-24574-4_28

[21] Sharan L, Burger L, Kostiuchik G, Wolf I, Karck M, De Simone R, Engelhardt S (2020) Domain gap in adapting self-supervised depth estimation methods for stereo-endoscopy. Curr Dir Biomed Eng 6(1). 10.1515/cdbme-2020-0004

[22] Sharan L, Romano G, Koehler S, Kelm H, Karck M, De Simone R, Engelhardt S (2021) Mutually improved endoscopic image synthesis and landmark detection in unpaired image-to-image translation. IEEE J Biomed Health Inform, p 1. 10.1109/JBHI.2021.3099858 10.1109/JBHI.2021.3099858 34310335

[23] Sofka M, Milletari F, Jia J, Rothberg A (2017) Fully convolutional regression network for accurate detection of measurement points. In: Cardoso MJ, Arbel T, Carneiro G, Syeda-Mahmood T, Tavares JMR, Moradi M, Bradley A, Greenspan H, Papa JP, Madabhushi A, Nascimento JC, Cardoso JS, Belagiannis V, Lu Z (eds) Deep learning in medical image analysis and multimodal learning for clinical decision support, lecture notes in computer science, pp 258–266. Springer, Cham. 10.1007/978-3-319-67558-9_30

[24] Stern A, Sharan L, Romano G, Koehler S, Karck M, De Simone R, Wolf I, Engelhardt S (2021) Heatmap-based 2d landmark detection with a varying number of landmarks. In: Palm C, Deserno TM, Handels H, Maier A, Maier-Hein KH, Tolxdorff T (eds) Bildverarbeitung für die Medizin 2021—proceedings, German workshop on medical image computing, Regensburg, March 7–9, Informatik Aktuell, pp 22–27. Springer. 10.1007/978-3-658-33198-6_7

[25] Sun P, Min JK, Xiong G (2015) Globally tuned cascade pose regression via back propagation with application in 2d face pose estimation and heart segmentation in 3d CT images. CoRR abs/1503.08843. http://arxiv.org/abs/1503.08843

[26] Yan Y, Naturel X, Chateau T, Duffner S, Garcia C, Blanc C (2018) A survey of deep facial landmark detection. In: RFIAP. Paris, France

[27] Yang J, Liu Q, Zhang K (2017) Stacked Hourglass network for robust facial landmark localisation. In: 2017 IEEE conference on computer vision and pattern recognition workshops (CVPRW), pp 2025–2033. IEEE, Honolulu, HI, USA. 10.1109/CVPRW.2017.253

[28] Zhang J, Liu M, Shen D (2017) Detecting anatomical landmarks from limited medical imaging data using two-stage task-oriented deep neural networks. IEEE Trans Image Process 26(10):4753–4764. 10.1109/TIP.2017.2721106 (Conference Name: IEEE Transactions on Image Processing) 10.1109/TIP.2017.2721106 PMC5729285 28678706

[29] Zhang J, Liu M, Wang L, Chen S, Yuan P, Li J, Shen SG, Tang Z, Chen K, Xia JJ, Shen D (2017) Joint craniomaxillofacial bone segmentation and landmark digitization by context-guided fully convolutional networks. In: Descoteaux M, Maier-Hein L, Franz AM, Jannin P, Collins DL, Duchesne S(eds) Medical image computing and computer assisted intervention—MICCAI 2017—20th international conference, Quebec City, QC, Canada, September 11–13, 2017, Proceedings, Part II, Lecture Notes in Computer Science, vol 10434, pp 720–728. Springer (2017). 10.1007/978-3-319-66185-8_81 10.1007/978-3-319-66185-8_81 PMC5786437 29376150

